# Performance of Community Water Board-Managed Passive In-Line Chlorinators Supported by a Circuit Rider Program in Rural Honduras

**DOI:** 10.1021/acsestwater.3c00425

**Published:** 2023-11-14

**Authors:** Megan Lindmark, Wesley Meier, Diana Calix, Craig Just

**Affiliations:** † Department of Civil and Environmental Engineering, University of Iowa, Iowa City, Iowa 52242, United States; ‡ EOS International, Saint Paul, Minnesota 55104, United States; § EOS International, Marcala, Honduras 15201, Central America

**Keywords:** chlorine, external support, circuit rider, community management, safe drinking water, passive chlorination, professionalized maintenance

## Abstract

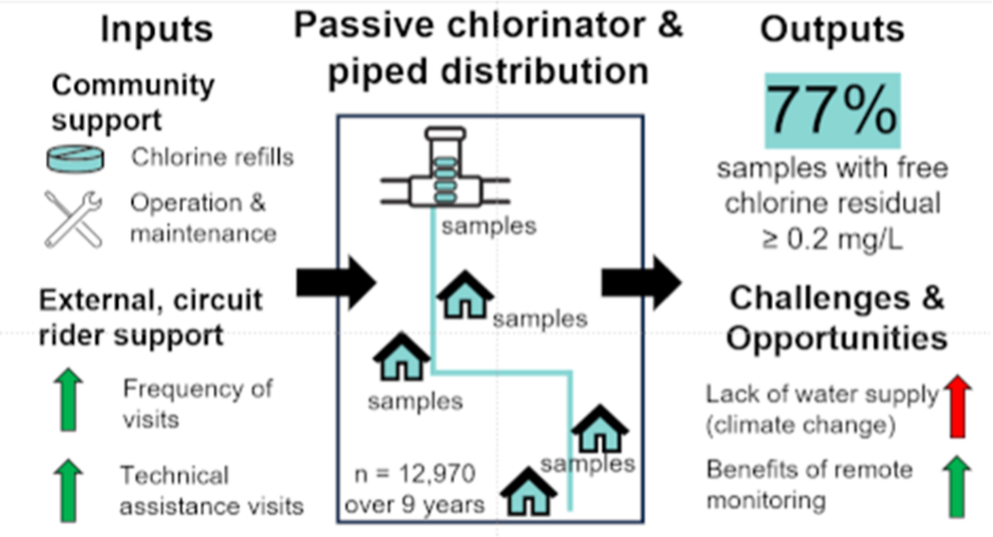

This study evaluated the ability of passive chlorinators and the associated kinds of external support necessary to provide adequate free chlorine residual (FCR) for community distribution systems in rural Honduras. We found that 77% of samples, from distribution systems with passive chlorinators installed by EOS International at storage tanks within these distribution systems, had FCR concentrations that met or exceeded the World Health Organization minimum threshold of 0.2 mg/L for point-of-use or piped systems. In EOS-supported communities, passive chlorinators delivered FCR ≥ 0.2 mg/L in 90% of tank samples, 83% of middle-house samples, and 79% of last-house samples. Technical issues accounted for only 26% of all lapses in chlorination (i.e., FCR = 0 mg/L). Occasional and habitual errors of the local water board accounted for 24 and 15% of all lapses. Visit frequency by EOS circuit riders was strongly correlated with positive chlorination outcomes, and technical assistance visits were the most valuable of all visit types. It was also shown that monitoring visits were negatively correlated with other visit types, indicating that monitoring may take place at the expense of more valuable visit types, which highlights the potential need for remote FCR monitoring approaches.

## Abstract 2

This study evaluated passive chlorinators in rural Honduras, the drivers of lapses in chlorination, and the types of circuit rider support necessary for sustained effectiveness.

## 1 Introduction

Two billion people lack access to safely managed drinking water, placing them at risk of consuming water with disease-causing pathogens.^1^ Providing water that is safely managed and therefore available on premises, when needed, and free of priority chemical and microbial contaminants is a priority of the UN sustainable development goals (SDGs). SDG 6, target 6.1, seeks universal access to safely managed drinking water for all by 2030.^2^ However, meeting this goal is unlikely since only 20% of countries yet to meet target 6.1 are on track to achieve safely managed drinking water for 100% of their population by 2030.^1^

Disinfection can provide water free of priority pathogens, and chlorine is the prominent disinfectant because it is accessible, affordable, can be used to treat water without electricity, and provides a free chlorine residual (FCR). Historically, chlorination as a household-level drinking water intervention has proven useful for emergency response, prolonged emergency settings, and community-level distribution systems.^3,4^ However, household-level options place the treatment burden on the individuals which can be ineffective^5,6^ and difficult to scale. Evidence from various community and institutional settings indicates that passive chlorinators are low-cost, scalable, adaptable, and can provide FCR concentrations meeting World Health Organization (WHO) guidelines.^7^ However, the effectiveness of passive chlorinators is largely context- and device-specific. A 2022 review of passive chlorination recommended that existing passive chlorinator installations be evaluated for evidence of providing adequate FCR at the point-of-collection (community shared taps or household distribution system connections) and for long-term effectiveness and site-specific maintenance requirements, particularly so that passive chlorinators can be recommended and implemented at scale.^7^

In the absence of ongoing support, community-level systems that provide improved access and/or water treatment are notorious for breakdowns and lapses in functionality.^8^ External support programs through NGOs and governments, can maintain and improve the functionality and financial management of community drinking water systems.^8−10^ Yet only one external support program included in a review specifically improved the microbial contamination of drinking water, primarily because very few external programs emphasize management of water quality.^8^ Unfortunately, evaluations to date rarely indicate which components and types of support have the strongest influence on improved access to safely managed drinking water.^9^ This is further compounded by the fact that most drinking water treatment evaluations in lower- and middle-income countries are short-term, and the support programs evaluated are a research component or are not sustained post-evaluation. Very few formal evaluations exist of sustained programs seeking to provide access to safely managed drinking water, but those that do emphasize the importance of professional evaluation of programmatic monitoring data.^11−13^ Specifically, no studies on passive chlorinators or external support programs for drinking water have focused on the kinds of support necessary to sustain effective water treatment by passive chlorinators.

In Latin America and the Caribbean, 25% of all households, and 47% of rural households, lack access to safely managed drinking water.^2^ In Honduras, where approximately 50% of the population lives in rural settings,^14^ 81% of rural households lack access to safely managed drinking water.^1^ Therefore, this study determined the technical and human factors that most influenced community-scale, passive chlorinator performance, and the circuit rider^15,16^ support types that improved outcomes in rural Honduras. We analyzed FCR measurements and survey data collected by EOS International circuit riders between 2013 and 2021 to (1) evaluate passive chlorinator capacity to maintain FCR ≥ 0.2 mg/L in the storage tank and distribution system; (2) determine specific technical failures and human errors associated with FCR < 0.2 mg/L; and (3) evaluate relationships between circuit rider visit periodicity, support types, and FCR.

## 2 Materials and Methods

### 2.1 Study Area and EOS International

We assessed passive chlorinator performance by evaluating FCR data from 359 communities (∼110,000 people) within the Comayagua, Copan, Intibucá, La Paz, Lempira, and Valle departments of Honduras (Figure S1) collected by circuit riders between January 2013 and December 2021. The communities were mostly rural with passive chlorinators being the only form of centralized water treatment. Sources included groundwater (springs and wells) and surface water (lakes, streams, and rivers). Small diameter (≤3 in. or 7.6 cm) PVC or steel pipes conveyed untreated source water to storage tanks (35 m^3^ average capacity) where the passive chlorinators were installed at the inlet. The storage tanks were typically connected to a piped distribution system with direct household connections, serving communities with an average population of 435. The sizes of the distribution systems were extremely variable, ranging from 2 to 200 km^2^.

EOS International, a nongovernmental organization based in El Salvador, Honduras, and Nicaragua, helps communities install passive chlorinators as part of their mission to empower rural communities to manage and maintain safe drinking water systems. EOS partners directly with community water boards, which in Honduras are nationally recognized legal entities^17^ mandated to manage and chlorinate community water supplies. Post-installation, EOS provides monitoring and technical support to community water boards through their circuit rider program. Circuit rider technicians make visits to communities monthly to deliver program elements. The EOS circuit rider program emphasizes monitoring, technical assistance, partnership with and training of community water boards, and the establishment of regional chlorine tablet distribution centers.

### 2.2 Passive Chlorinator Installation, Management, and Community Engagement

**Figure 1:**
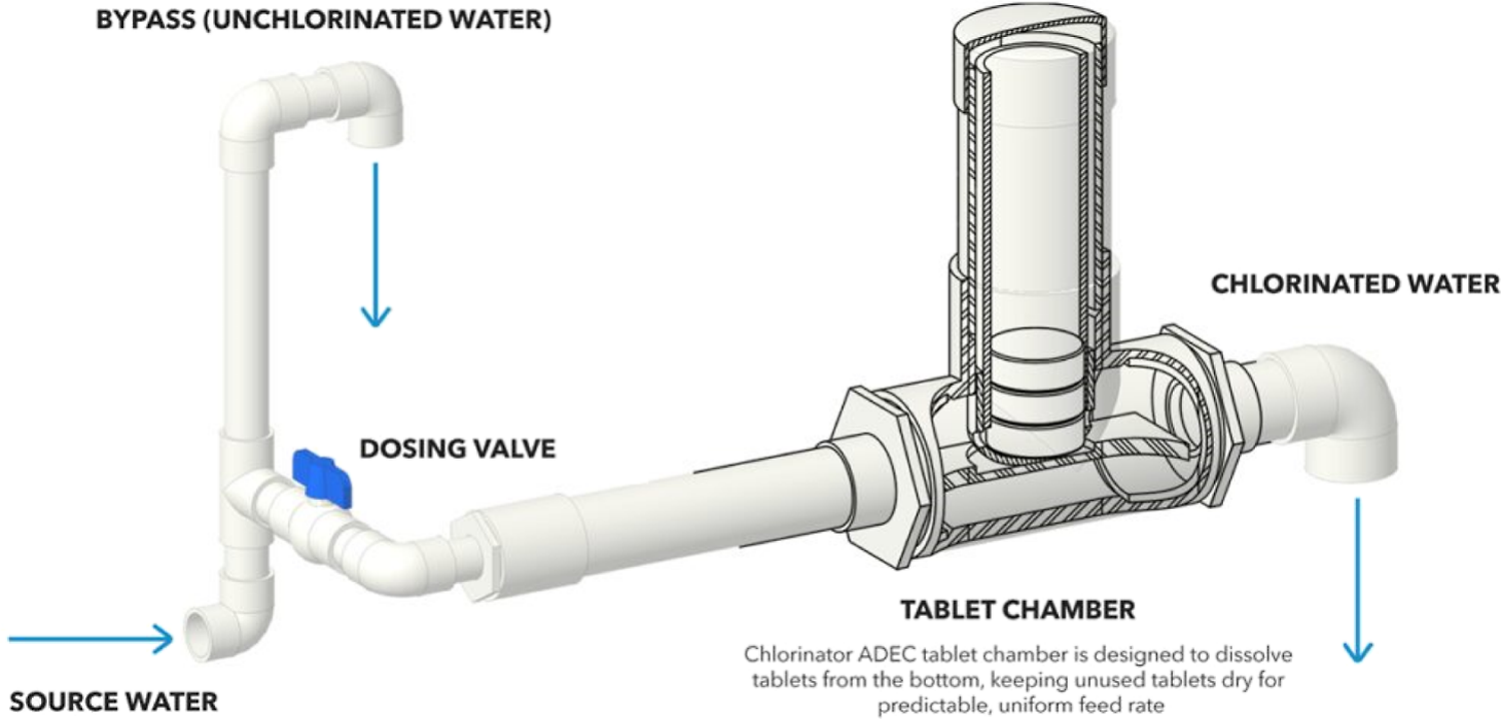
Clorador ADEC design, indicating flow of chlorinated and unchlorinated water and internal design of the tablet chamber.

Two similar passive chlorinators, the Compatible Technology International (CTI) 8 and the Clorador ADEC (Agua de Calidad), were in use during the study period (Figure 1). Both passive chlorinators used 2–5/8 to 3 in. (6.7 to 7.6 cm) diameter calcium hypochlorite tablets and were typically installed on top of a water storage tank and piped into the tank inlet. Immediately after installation and during technical assistance visits as needed, circuit riders coarsely adjusted the FCR dose at the tank, to a target of 1.5–2.5 mg/L, using the valve that controlled the flow rate through the tablet chamber. Both passive chlorinators were designed to operate at flow rates between 2 and 20 gallons per minute (7 to 76 LPM) but could be altered or installed in parallel to serve communities with greater flow rates.

**Figure 2:**
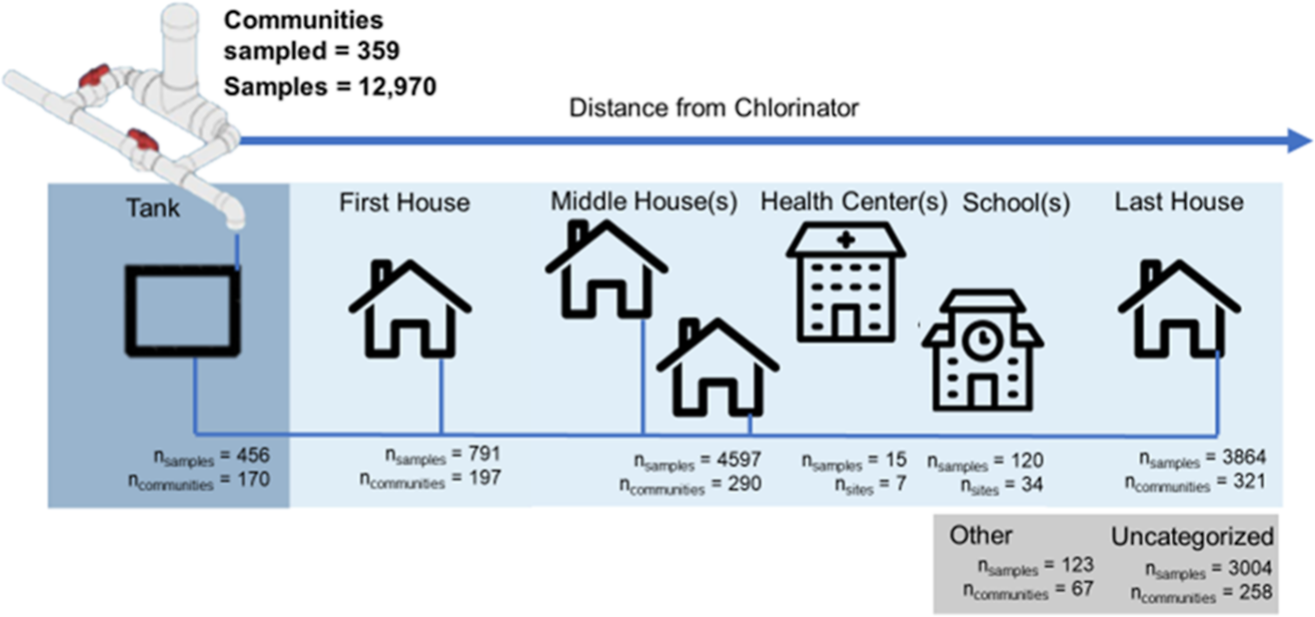
Distribution of sampling locations beginning at the tank (point of treatment) directly following each chlorinator. Additional and uncategorized sampling sites may be anywhere within this distribution system (point of collection).

EOS circuit riders visited communities monthly and, on each visit, surveyed water board members and measured the FCR via the *n*-diethyl-*p*-phenylenediamine (DPD) method (chlorine, low range 8021, Hach, Loveland, CO; free chlorine, 3308-01, LaMotte, Chestertown, MD).^18^ Sampling points included the storage tank, first house, middle house, and last house connected to the distribution system (Figure 2). In addition, water samples from selected schools and health centers within the distribution system were collected and analyzed. When inadequate FCR was measured, circuit riders determined the cause, assigned primary and secondary failure type(s), and implemented a response (Table 1). In addition to monitoring, EOS circuit riders implemented 10 other visit types, including technical assistance, special events, and training around themes such as sustainable tariff collection, board management, source protection, chlorination, and more (Table 2), during the study period, which included 18,722 total visits and 12,970 FCR monitoring visits.

**Table 1:** Types of Lapses in Chlorination, Definitions, and Circuit Rider Responses

lapse type	definition	circuit rider response
Technical−System Error		
insufficient water	no water flowing through the system	determine cause
chlorinator	water and chlorine tablets present, but no free chlorine residual	meet with the water board, recalibration of chlorinator valves (if necessary)
		
Human Error		
occasional error of the community water board in replacing chlorine	community occasionally but rarely forgets to replace chlorine tablets (as defined by EOS circuit riders)	alert water board junta leader
habitual error of the community water board in replacing chlorine	community habitually forgets to replace chlorine tablets	schedule follow-up training for the water board
inactive community water board	community management board is inactive	not applicable
no chlorine tablets purchased	no chlorine tablets were available for replacement in the chlorinator	alert water board leader, determine the reason for no chlorine stock (i.e., cost)
		
Other		
repairs	ongoing plumbing repairs or repairs needed before chlorination can continue	not applicable
pump	failure of water pump feeding influent	determine cause
turbidity	turbidity was too high, and chlorination was halted for safety	not applicable
unlisted	no reason for failure listed	not applicable

**Table 2:** Circuit Rider Type and Definitions

visit type	definition
chlorine monitoring	visit to perform monthly chlorine monitoring
technical assistance	visits for circuit riders to provide technical assistance to the community water board (i.e., flow rate measurement, calibration, inspection, installation of components, etc.)
training	training conducted by circuit riders for members of the water board on system management. Topics include water board administration, chlorination, plumbing, water governance, watershed protection, operation and maintenance, community organization, and tariffs and financial management
development of new projects	meetings to develop new projects or possible chlorinator installation locations
chlorine entry	delivery of chlorine tablet supply at the community or nearby chlorine bank
special event	special events with community members or water board members
installation	installation of chlorinator
office	office or administrative-related visits
construction of chlorinator	construction, repair, or update of PVC chlorinator
meeting	a meeting between a circuit rider and community members or water board members
other	community visits that do not otherwise fit into these categories

### 2.3 Data Collection and Management

All survey data were entered into the online, open-access, data management platform, mWater.^19^ We compiled the data set by exporting mWater surveys between 2013 and 2021 for the communities located in the study area and additional community descriptors such as population, source type, water supply type, tank size, department, and municipality were retrieved from the EOS community profile surveys (Table S1). We removed data associated with chlorine banks, communities without mWater identification numbers, and monitoring visits without FCR measurements resulting in the exclusion of 145 monitoring visit data values and 1644 other visit-type entries. When sample location or failure type data were missing, we assigned values derived from the circuit rider site descriptions whenever possible and “other” when not possible. For instances involving multiple failure types, only the primary failure type was considered.

### 2.4 Data Analysis

We used the D’Agostino-Pearson test to evaluate the normality of our data. We used the Kruskal–Wallis test and Dunn’s multiple comparison test to compare median chlorine concentrations for each of the categories within sample location, year, department, and source water type (GraphPad Prism 9.0, La Jolla, CA). We used the WHO piped water and household point-of-use FCR recommendations^20^ of 0.2–0.5 and 0.2–2.0 mg/L, respectively, to inform the “adequate FCR” level of ≥0.2 mg/L used in our analysis. To evaluate changes in the FCR between 2013 and 2021, we categorized annual FCR data as 0–49, 50–74, 75–99, or 100% of samples ≥0.2 mg/L for each community. We also categorized the lapses in FCR between 2015 and 2021 as either human error- or technical failure-related (Table 1) before calculating the percentage of each lapse type relative to the total number of monitoring visits performed.

To determine the impact of different types of support visits and their periodicity, we evaluated the relationship between various types of community visits and the mean FCR concentration. We used the Psych^21^ and corrplot^22^ R packages to apply the Spearman nonparametric correlation test (*p* < 0.05) with Bonferroni corrections (*p*_adj_ < 0.005) and to plot the corresponding correlation grids. To calculate the percentage of possible visits, we assumed visits could only be completed monthly and divided the number of actual visits by the age of the system (in months).

## 3 Results

**Figure 3:**
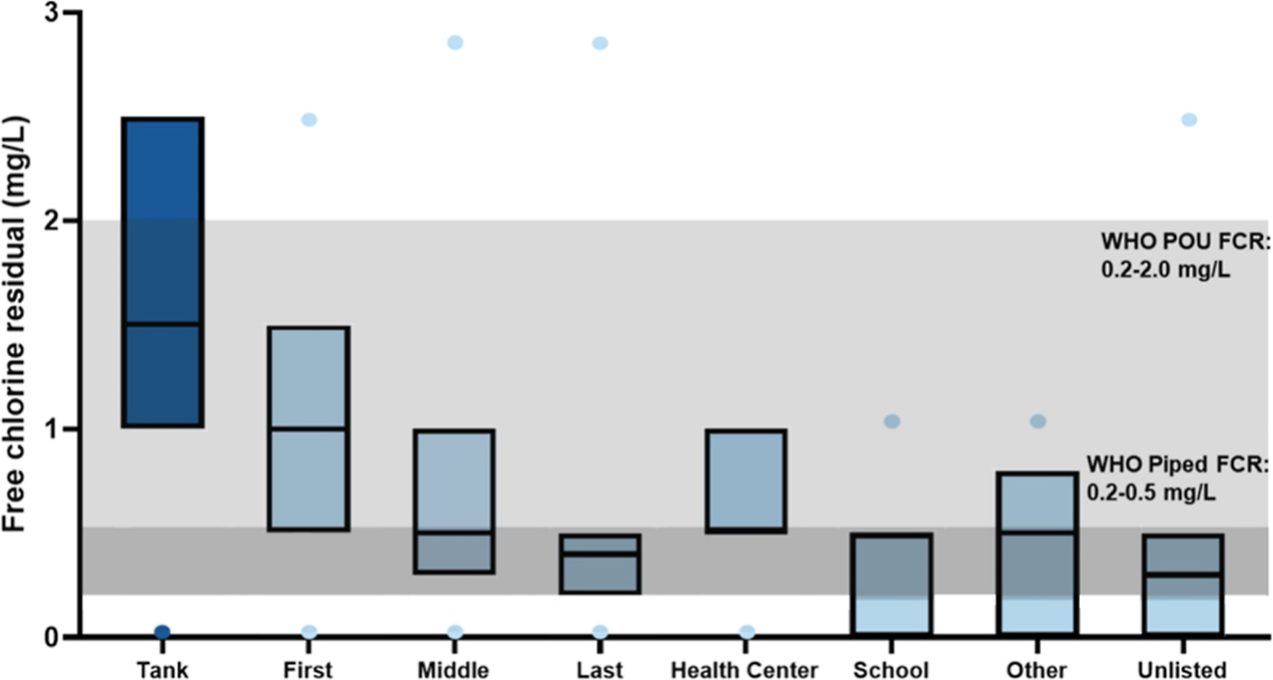
FCR concentrations from tanks and points-of-collection. The dots above or below each box represent minima or maxima, and the top, bottom, and internal lines of each box represent the 75th percentile, 25th percentile, and median, respectively. The grayscale shading represents the WHO point-of-use and piped water FCR guidelines.

Seventy-seven percent of all samples collected from the tanks (point of treatment) or distribution systems (point-of-collection) between 2013 and 2021 had FCR ≥ 0.2 mg/L (Table S2). The percentage of samples with FCR ≥ 0.2 mg/L increased from 61% in 2013 to 76% in 2021, with a maximum of 86% in 2020. The median FCR of 1.5 mg/L at the tank was well above WHO guidelines and was statistically greater than the median for samples obtained from the point-of-collection (Figure 3, Tables S2, S3), except for health centers, where the small sample size resulted in low statistical power. As expected, the median FCR for point-of-collection samples was highest at the first house(s) and decreased as the distance from the tank increased, but all median values across the distribution system were >0.2 mg/L. Systems fed by groundwater had a higher median FCR than samples from surface-fed and unlisted source-type systems (Figure S2). During the study period, the percentage of communities with 100% of annual samples having sufficient FCR increased from 2% in 2013 to 57% in 2021 (Figure S3).

**Figure 4:**
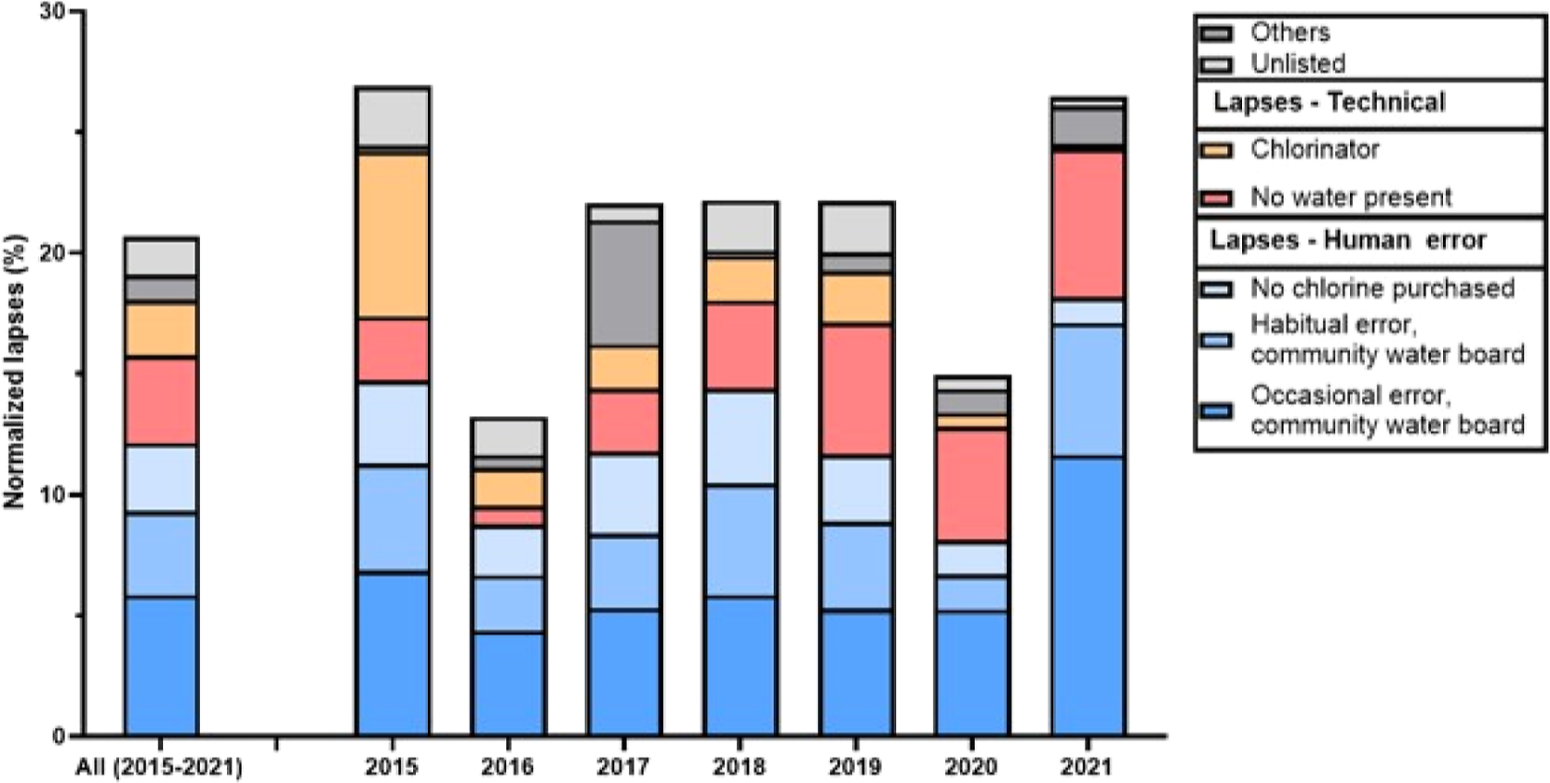
Annual percent of technical failures and human errors associated with FCR = 0 mg/L normalized to the number of samples collected each year. The other category includes repairs, pump failures, and high turbidity.

Human error caused the most lapses in chlorination (FCR = 0 mg/L) for all visits from 2013 to 2021 and accounted for the largest percentage of samples with no chlorine in every individual year (Figure 4). Occasional and habitual errors of the water board accounted for 24 and 15% of all lapses, respectively. Lapses attributed to the water board forgetting or lacking sufficient resources to purchase chlorine accounted for 11% of all lapses. Technical issues accounted for only 26% of all lapses in chlorination and were delineated as system-level or chlorination-specific. System-level challenges include a lack of flowing water, pump failures, and ongoing repairs. Chlorination-specific lapses include failure of the chlorinator and intentional halting of chlorination due to high turbidity. Technical lapses attributed to the chlorinator decreased from 25% in 2015 to less than 1% in 2021, and turbidity caused only 0.3% of the lapses between 2015 and 2021. The percentage of lapses attributed to the lack of flowing water increased each year between 2016 and 2021 and accounted for 21% of lapses in 2021.

**Figure 5:**
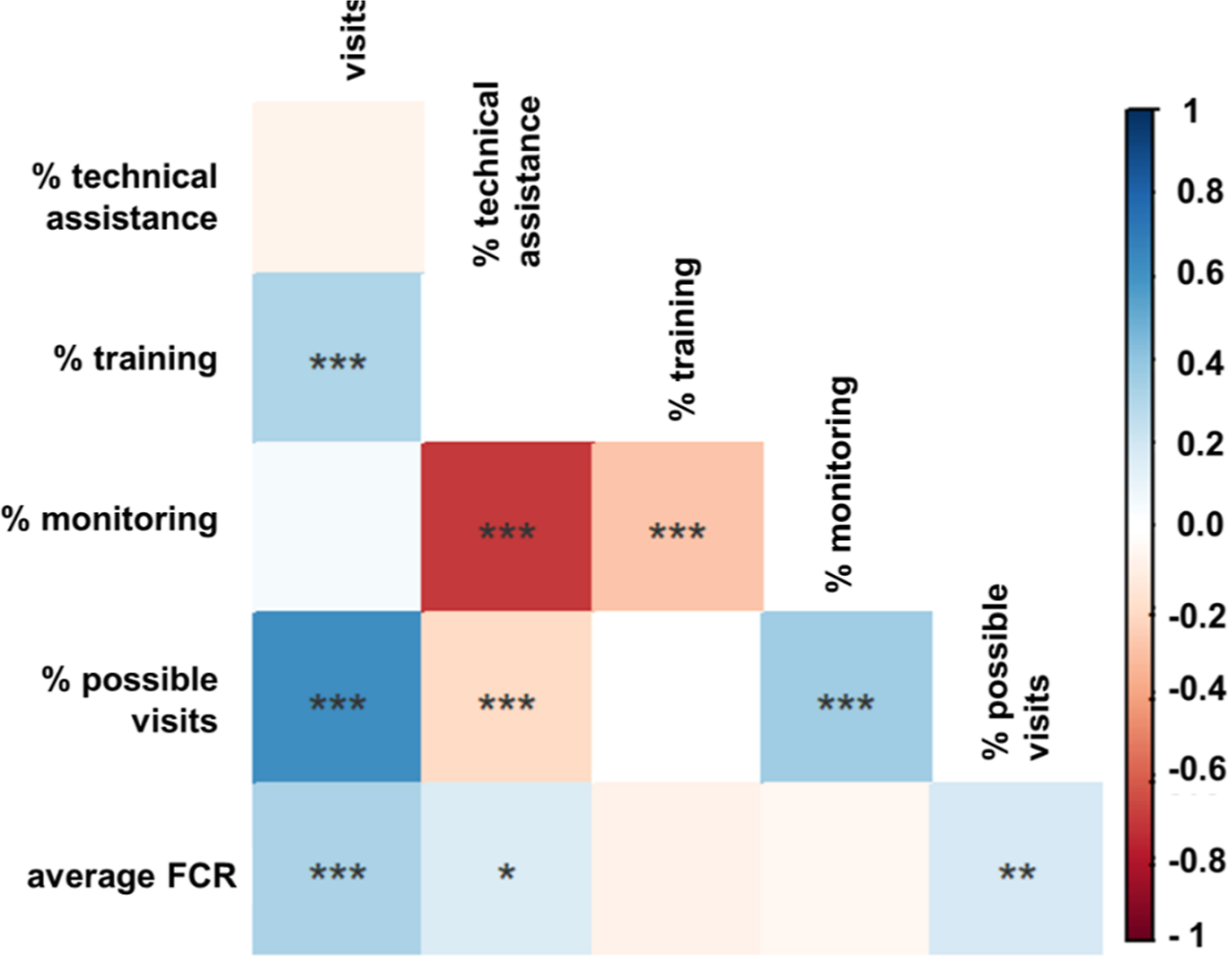
Spearman correlations between circuit rider visit periodicity, support types, and average FCR. Adjusted p-values: * < 0.0045, ** < 0.0009, *** < 0.000091. Additional correlation coefficients and *p*-values are available in the Supporting Information (Figure S4).

To evaluate the outcomes of visit type on average FCR, we performed a series of pairwise Spearman correlations (Figure 5) that yielded coefficients indicating the strength of the resulting monotonic relationships. The most strongly, negatively correlated pairing was the percentage of monitoring visits and technical assistance visits (Spearman *r* = 0.71, *p*_adj_ < 0.0045). The percentage of training visits was also negatively correlated with the percentage of monitoring visits. This indicates that increased monitoring visits likely consumed time that could have been used to perform additional training and technical assistance visits. Except for the technical assistance visits (Spearman’s *r* = 0.17, *p*_adj_ < 0.0045), no individual visit type had a strong relationship with FCR. However, the absolute number of visits was positively correlated with the average FCR (Spearman *r* = 0.321, *p*_adj_ < 0.0045). Furthermore, system age was positively correlated with average FCR (Spearman *r* = 0.27, *p*_adj_ < 0.0045) and the number of visits (Spearman *r* = 0.6, *p*_adj_ < 0.0045). However, we were unable to determine whether it was simply age that was strongly related to average FCR or if the increased age of the system allows for additional visits, which strongly relates to average FCR. The percentage of possible visits completed was also positively correlated with the average FCR (Spearman *r* = 0.19, *p*_adj_ < 0.0045), which indicates that both the number and consistency of visits over time positively impact FCR.

## 4 Discussion

The passive chlorinators, supported by EOS between 2013 and 2021, maintained FCR ≥ 0.2 mg/L in 77% of the samples collected at the point of treatment and point of collection. This effectiveness matched or exceeded tablet-based passive chlorinators of a similar design.^15,23,24^ Specifically, EOS passive chlorinators had higher rates of effective chlorination at the point of collection compared to similar contexts.^15^ Self-constructed tablet passive chlorinators in Honduras, evaluated by Henderson et al., yielded an FCR ≥ 0.2 mg/L in 90% of the tank samples, 41% of the middle house samples, and 31% of the last house samples.^15^ In EOS-supported communities, passive chlorinators delivered FCR ≥ 0.2 mg/L in 90% of tank samples, 83% of middle house samples, and 79% of last house samples (not including schools, health centers, unlisted, or other categories of sample location). Notably, the comparatively long timespan (9 years) and the large number of communities (*n* = 359) in our evaluation make it the longest and largest evaluation of passive chlorinator effectiveness. Our 2022 review of passive chlorinators noted that there is a paucity of long-term evaluations of sustained performance of passive chlorinators in communities, outside of typical short-term evaluation periods; a critical gap that our evaluation begins to fill.

Adoption of chlorination, at the household level, requires continued intervening support.^25^ However, this relationship and, specifically, the sustained effectiveness of chlorination is not well evaluated when chlorination occurs at the community level, and adherence must be maintained by an elected board of water managers. Our results indicate that the leading driver of lapses in chlorination are choices made by the water board, specifically the lack of action on the part of the water board. Habitual human errors caused 15% of all chlorination lapses observed in this study. The occasional lack of tablet replacement by the water board accounted for the largest percentage of chlorination lapses. Although this type of lapse was less likely to be repeated than habitual errors, even infrequent interruptions to chlorination can drastically minimize the positive health benefits of safe drinking water.^26^ Our study provides anecdotal evidence of occasional tank chlorination cessation when large amounts of water were used for adobe-style home construction or for washing coffee beans. A Global Brigades report found that many coffee farmers prefer untreated water to avoid perceived negative consequences.^27^ However, there is no scientific evidence of the impact of chlorination on coffee production. Therefore, temporary cessation of chlorination due to coffee production represents an important challenge for circuit riders to specifically address in technical assistance and training visits to communities.

Although lapses in chlorination attributed to water board failure to purchase the chlorine tablets required to replenish the passive chlorinators made up the smallest proportion of human error-related lapses, it is still critical to examine the possible reasons underlying these lapses. Evaluations conducted on other passive chlorinators in Nepal and Uganda indicated that supply chain challenges can limit chlorine replacement availability.^6,24^ However, the EOS program includes chlorine tablet distribution across Honduras to local chlorine banks, and communities can purchase tablets directly from the EOS offices and circuit riders. Therefore, the community water board’s failure to purchase chlorine tablets may be related to affordability. Water boards rely on water tariff fees paid by community members to purchase chlorine tablets, and lack of payment could result in insufficient funding. Affordability and willingness to pay for chlorine refills could not be evaluated fully with the data curated for this study. Other evaluations have measured willingness to pay for passive chlorinators in Bangladesh^28^ and Kenya^29^ and can guide future methodology, but context-specific willingness to pay for chlorine refills should, therefore, be a focus of future work.

The periodicity of visits conducted by EOS circuit riders was positively correlated to the outcome FCR, indicating that communities more frequently visited experienced improved access to safely managed drinking water. This corroborates evidence that circuit rider programs can decrease instances of microbiologically contaminated drinking water^9,30^ and, given the dearth of circuit rider programs focused holistically on water supply and quality, provides a rationale for scale-up. Further evidence for scale-up can be found in professionalized maintenance arrangements “where legal and regulated service providers perform preventive maintenance and repairs for water supply infrastructure in exchange for payment to achieve pre-determined service outcomes”.^31^ The EOS circuit rider program resembles professionalized maintenance agreements which are increasingly being implemented to service hand pumps and water points in Sub-Saharan, Africa^31^ to improve system uptime.^16,32^ These results further suggest that the kinds of behavioral nudges such as reporting of chlorine results that can encourage adherence to household chlorination^25^ can encourage chlorination at the community level. Nowicki et al.,^33^ in 2022, found that sharing *E. coli* results with water managers in rural communities in Kenya motivated them to respond proactively, mitigate potential contamination, and manage water to avoid future positive tests. Similarly, our study showed that FCR was positively correlated with the number of support visits completed, which suggests that oversight and exposure to monitoring results as reported by EOS circuit riders to water board members can increase average FCR. But perhaps more importantly, although visits alone provide a benefit, the type of support provided during those visits is the most critical for improved outcomes. Our results also indicate that technical assistance visits were most positively correlated with outcome FCR, a correlation shared with no other visit types. Furthermore, technical assistance visits were negatively correlated with other visit types. This suggests that finite circuit rider visit time should be devoted to technical assistance and that other visit types, such as monitoring, should increasingly be accomplished by other means when possible.

Our evaluation also showed that lapses attributable to a lack of flowing water are increasing. Some distribution systems in our study were over 20 years old, often much older than the passive chlorinators themselves.^14^ Distribution system performance may be problematic because aging infrastructure can be correlated with breakdowns and system downtime.^34^ In cases when a decrease or total loss of source flow caused more frequent lapses, we suggest that climate change may be the underlying reason and drinking water-associated health outcomes may be compromised as a result.^35,36^ The 2021 Intergovernmental Panel on Climate Change (IPCC) regional report^37^ indicated that Central America is experiencing increased water variability attributable to more intense and more frequent droughts and rainfall, and the magnitude of this variability is expected to increase.^37^ Without a primary drinking water source, community members are more likely to use less safe, unchlorinated sources, which can negate many of the positive health benefits^26^ associated with passive chlorinators.

We recommend that EOS find innovative ways to focus their circuit rider program on providing the technical assistance visits that most strongly correlate to improved outcomes. For example, sensor-based monitoring of water, sanitation, and hygiene (WASH) interventions is increasing and has the potential to offset personnel requirements.^38,39^ Sensors could be deployed with passive chlorinators to monitor source water availability and FCR and provide alarms to trigger technical support visits by circuit riders. Additionally, ongoing efforts by EOS to transfer the responsibility of monitoring to key community members, community health center volunteers, or municipal government entities in communities with strong records of chlorination could further optimize personnel time. If monitoring and reporting requirements can be successfully transferred to the communities with continued guidance, necessary supplies, and support from EOS, then EOS circuit riders could prioritize technical assistance visits for community water systems in disrepair while continuing to expand passive chlorinator installations into new communities. Over 321 million people in Latin America and the Caribbean, and 2.32 billion people globally, are served by drinking water systems compatible with passive chlorinators.^40^ Therefore, we assert that passive chlorinators coupled with an external circuit rider support program, like the one described here, can be replicated at scale to accelerate progress toward safely managed drinking water for all.

## 5 Limitations

The data set used for the analysis in this study was collected by various EOS circuit riders over the study period, initially via paper surveys, then via Excel documentation, and now in the form of mWater mobile-based surveys. The thousands of data points gathered throughout the course of this study may have included errors, but we believe that our quality assurance measures adequately addressed this potential shortcoming.

## 6 Conclusions

This study determined the technical and human factors that most influenced community-scale, passive chlorinator performance, and the circuit rider support types that improved outcomes in rural Honduras. Our evaluation of passive chlorinator capacity to maintain FCR ≥ 0.2 mg/L in storage tanks and distribution systems showed this minimum threshold was met by 77, 90, 83, and 79% of all, storage tank, middle house, and last house samples, respectively. Our determination of specific human errors and technical failures associated with FCR < 0.2 mg/L showed that local water board errors such as occasional, habitual, and cost-associated chlorination lapses accounted for 24, 15, and 11% of all lapses, respectively. Technical failures accounted for 26% of all lapses and chlorinator-related lapses decreased from 25% in 2015 to less than 1% in 2021. Source water availability technical lapses increased since 2016 to 21% in 2021.

Overall, our evaluation of relationships between circuit rider visit periodicity, support types, and FCR revealed that lapses in chlorination or instances when water did not meet WHO standards were predominantly caused by local water boards forgetting or choosing not to replenish chlorine tablets. However, we confirmed that external support, coupled with local community water board operation and management, was necessary to sustain adequate chlorine concentration and counter these lapses. Specifically, the frequency of visits by EOS circuit riders was strongly correlated to positive chlorine outcomes, particularly when those visits provided technical assistance to community water boards to support operation and maintenance. Overall, our results demonstrate that community-managed passive chlorinators, coupled with specific and frequent external support, provide adequate FCR as one component for safely managed drinking water. Our findings inform future passive chlorinator implementations by identifying the types of ongoing operation and maintenance support necessary to ensure sustained effectiveness and positive health outcomes.

## Supporting Information Available

Additional results including significance values for comparisons, summary medians and IQRs, figures demonstrating annual changes, difference between ground and surface water, and tables showing survey elements collected from mWater (PDF)
