# Supplementary material for: Performance of Community Water Board-Managed Passive In-Line Chlorinators Supported by a Circuit Rider Program in Rural Honduras
Source: ACS ES T Water. 2023 Nov 14;3(12):4011–9. doi: 10.1021/acsestwater.3c00425 (PMC10714393; doi:10.1021/acsestwater.3c00425)
Supplement: Supplementary file 1 — ew3c00425_si_001.pdf [file ew3c00425_si_001.pdf]

Supporting Information (SI)

Manuscript Title: **Performance of community water board-managed passive in-line chlorinators supported by a circuit rider program in rural Honduras**

Megan Lindmark[1,2*], Wesley Meier[2], Diana Calix[3], Craig Just[1]

Affiliations:

1. Department of Civil and Environmental Engineering, University of Iowa, Iowa City, Iowa 52242, United States
2. EOS International, Saint Paul, Minnesota 55104, United States
3. EOS International, Marcala, Honduras 15201, Central America

*corresponding author, megan.lindmark@eosintl.org

Table of Contents

Figure S1. The study area 2

Table S1. Circuit rider program and community profile elements from the EOS mWater survey. 3

Table S2. Free chlorine residuals (FCRs) and interquartile ranges by year, sampling location, source type, and department. 4

Table S3. Significance values for the Kruskal Wallis ANOVA and Dunn's multiple comparisons tests. .. 5

Figure S2. FCR concentrations for tank and distribution system sampling points from surface and ground water sources........ 6

Figure S3. Annual percentage of communities maintaining FCR ≥ 0.2 mg/L for tank and point-of-collection samples. 7

Figure S4. Spearman correlations coefficients and adjusted p-values between circuit rider visit periodicity, support types, and average FCR. 8

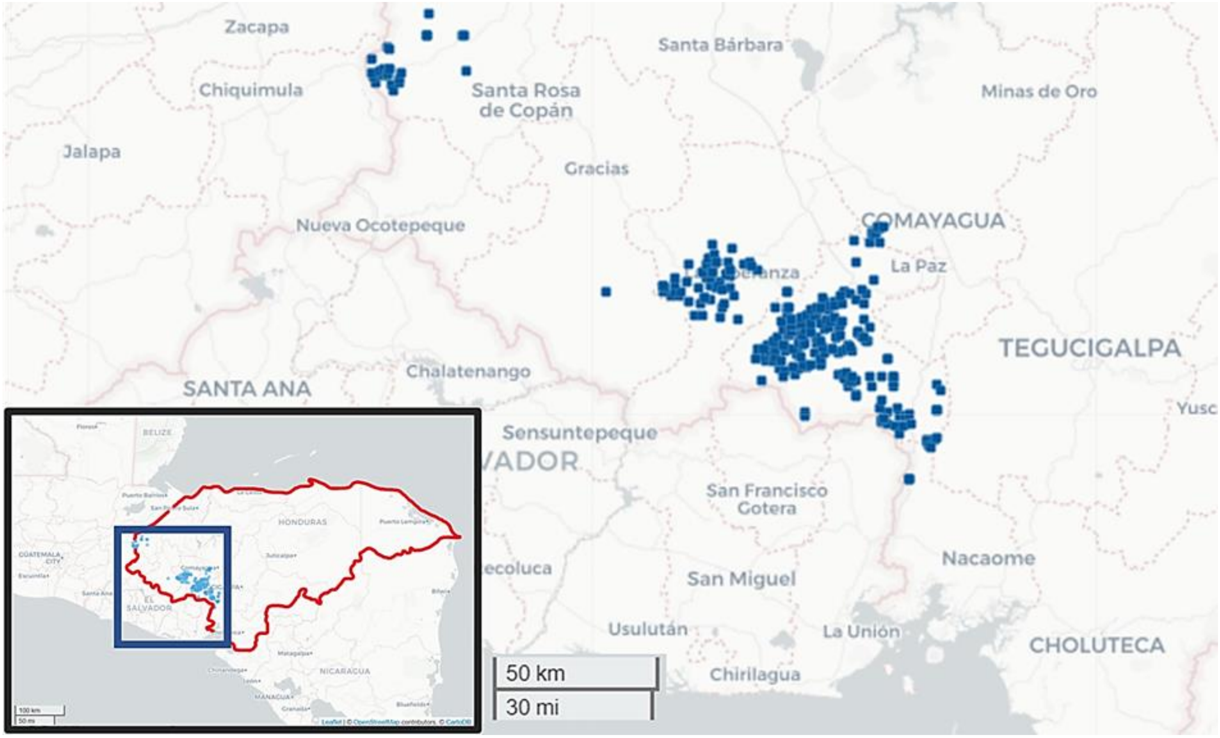

Figure S1. The study area included 359 chlorinators within rural communities across the Comayagua, Copan, Intibuca, La Paz, and Lempira departments of Honduras. Credit: mWater map generator (Feighery and Feighery 2020).

Table S1. Circuit rider program and community profile elements from the EOS mWater survey.

**Circuit rider survey elements**
1. General visit information (completed for all visit types): Country, Circuit rider, Visit date, Type of visit, Community or chlorine bank identification number
2. Chlorine monitoring visit: Chlorine presence/absence, Concentration, Sampling location, Problems y/n, Type(s) of problems, Additional details
3. Training visit: Type of training
4. Technical assistance visit: Type of technical assistance, Water sample collected y/n, Tank flow, Source flow, Monthly water tariff, Number of water system users
5. Office visit: Type of office visit, Additional details
6. Chlorine entry: Type of entry, Volume or weight of chlorine, Number of tablets, Number of buckets, Number of filters, Number of reagents, Current chlorine stock, Payment received
7. Installation visit: Type of installation, Photos
8. Meeting visit: Location type
9. Development of new projects: No other questions
10. Construction of chlorinator: No other questions
11. Other: More details
**Community profile survey elements**
1. Identifying information: Country, Community, Department, Municipality, Circuit rider, Activity level, Date of last activity check
2. Community photos: Water board, Tank, Chlorinator, Source
3. Water system information: Population, Chlorinator type, Installation date, Installation technician, Source type, Catchment type, Tank capacity, Community, school, or business, Chlorine bank

Table S2. Free chlorine residuals (FCRs) and interquartile ranges by year, sampling location, source type, and department.

	Communities	*Samples*		*FCR ≥ 0.2 mg/L*		*Median FCR (mg/L)*
	n	n	%	n	%	(IQR)
All	359	12,970	100	9982	77	0.5 (0.2,0.8)
Year						
2013	88	879	6.8	539	61.3	0.5 (0.2,0.7)
2014	91	976	7.5	684	70.1	0.4 (0.0,0.5)
2015	143	1628	12.8	1174	72.1	0.3 (0.0,0.5)
2016	150	1392	10.7	1180	84.8	0.5 (0.3,0.9)
2017	200	1035	8.0	791	76.4	0.5 (0.2,1.0)
2018	233	2408	18.6	1859	77.2	0.5 (0.2,1.3)
2019	271	1794	13.8	1400	78.0	0.5 (0.3,1.0)
2020	251	1805	13.9	1547	85.7	0.5 (0.5,0.8)
2021	231	1053	8.1	808	76.7	0.5 (0.2,0.6)
Sampling Location						
Tank	170	456	3.5	409	89.7	1.50 (1.0,2.5)
First house	197	791	6.1	660	83.4	1.00 (0.5,1.5)
Middle house	290	4597	35.4	3798	82.6	0.50 (0.3,1.0)
Last house	321	3864	29.8	3066	79.4	0.40 (0.2,0.5)
Health center	7	15	0.12	13	86.7	0.50 (0.5,1.0)
School	34	120	0.93	87	72.5	0.50 (0.0,0.5)
Other	67	123	0.95	92	74.8	0.50 (0.0,0.8)
Unlisted	258	3004	23.2	1857	61.8	0.30 (0.0,0.5)
Source Type						
Spring	77	3129	24.1	2714	86.7	0.50 (0.5,0.8)
Lake	9	570	4.4	422	74.0	0.50 (0.1,0.5)
Stream	141	5790	44.6	4439	76.7	0.50 (0.2,0.7)
River	20	522	4.0	376	72.0	0.50 (0.0,0.1)
Well	3	156	1.2	129	82.7	0.50 (0.3,1.0)
Unlisted	109	2803	21.6	1902	67.9	0.50 (0.0,1.0)
Department						
Comayagua	10	474	3.6	409	86.3	0.70 (0.5,1.4)
Copan	31	727	5.6	417	57.4	0.50 (0.0,1.5)
Intibuca	84	3229	24.9	2813	87.1	0.50 (0.5,0.8)
La Paz	225	8482	65.4	6302	74.3	0.50 (0.1,0.7)
Lempira	5	12	0.09	12	100	0.50 (0.2,0.5)
Valle	4	46	0.35	29	63.0	0.20(0.0,0.5)

Table S3. Significance values for the Kruskal Wallis ANOVA and Dunn's multiple comparisons tests.

Dunn's multiple comparisons test	Mean rank diff.	Summary	Adjusted P Value
Tank (n=447) vs. First (n=761)	1897	***	<0.0001
Tank (n=447) vs. Middle (n=4479)	3149	***	<0.0001
Tank (n=447) vs. Last (n=3542)	5093	***	<0.0001
Tank (n=447) vs. Health Center (n=15)	2913	ns	0.0501
Tank (n=447) vs. School (n=120)	5042	***	<0.0001
Tank (n=447) vs. Other (n=123)	4125	***	<0.0001
Tank (n=447) vs. Unlisted (n=3004)	5230	***	<0.0001
First (n=761) vs. Middle (n=4479)	1252	***	<0.0001
First (n=761) vs. Last (n=3542)	3196	***	<0.0001
First (n=761) vs. Health Center (n=15)	1017	ns	>0.9999
First (n=761) vs. School (n=120)	3145	***	<0.0001
First (n=761) vs. Other (n=123)	2228	***	<0.0001
First (n=761) vs. Unlisted (n=3004)	3334	***	<0.0001
Middle (n=4479) vs. Last (n=3542)	1944	***	<0.0001
Middle (n=4479) vs. Health Center (n=15)	-235.4	ns	>0.9999
Middle (n=4479) vs. School (n=120)	1893	***	<0.0001
Middle (n=4479) vs. Other (n=123)	975.7	ns	0.0747
Middle (n=4479) vs. Unlisted (n=3004)	2081	***	<0.0001
Last (n=3542) vs. Health Center (n=15)	-2179	ns	0.4986
Last (n=3542) vs. School (n=120)	-50.66	ns	>0.9999
Last (n=3542) vs. Other (n=123)	-968.0	ns	0.0835
Last (n=3542) vs. Unlisted (n=3004)	137.6	ns	>0.9999
Health Center (n=15) vs. School (n=120)	2128	ns	0.8053
Health Center (n=15) vs. Other (n=123)	1211	ns	>0.9999
Health Center (n=15) vs. Unlisted (n=3004)	2317	ns	0.3302
School (n=120) vs. Other (n=123)	-917.3	ns	>0.9999
School (n=120) vs. Unlisted (n=3004)	188.3	ns	>0.9999
Other (n=123) vs. Unlisted (n=3004)	1106	*	0.0202

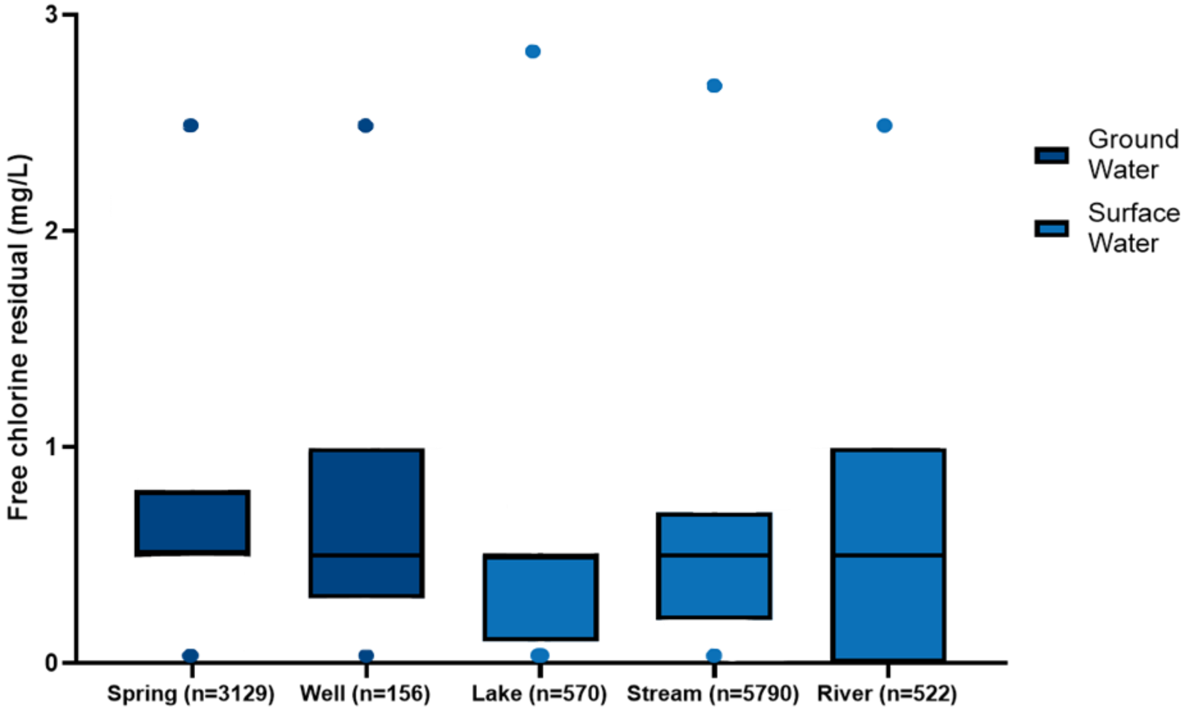

Figure S2. FCR concentrations for tank and distribution system sampling points from surface and ground water sources. The dots above or below each box represent maximum or minimum values. The top, bottom, and internal line of each box represent the 75th percentile, 25th percentile, and the median, respectively. Samples with unlisted source water (n=2803) were excluded.

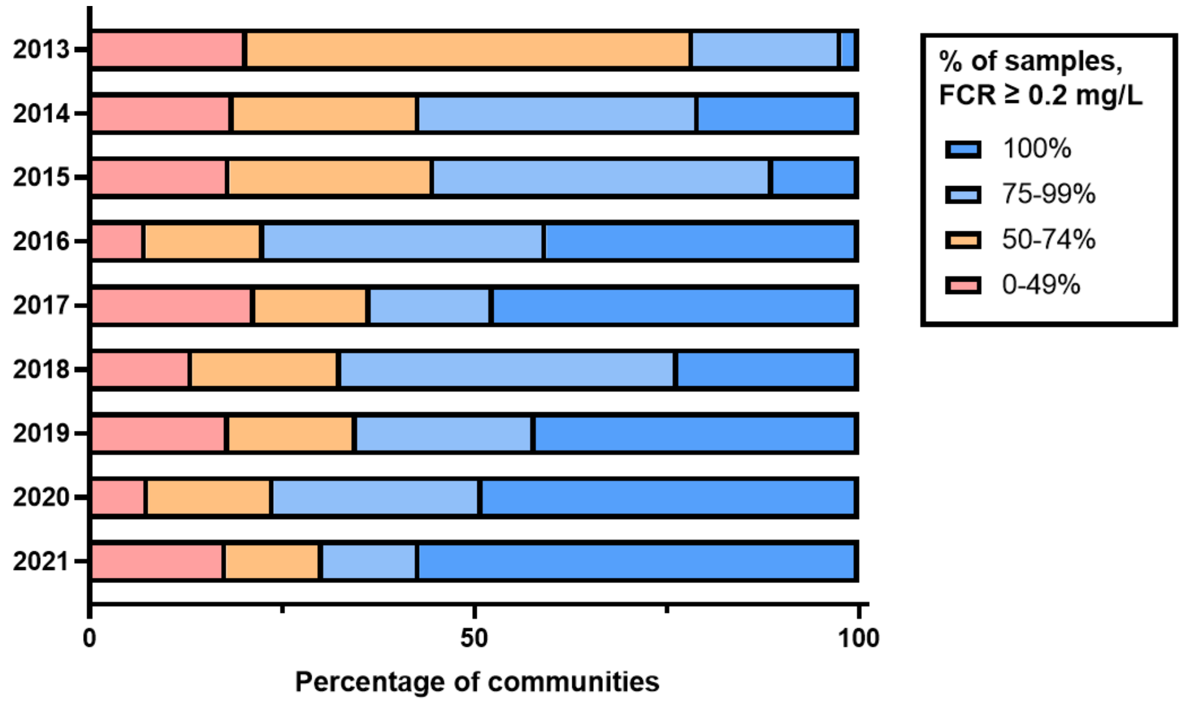

Figure S3. Annual percentage of communities maintaining FCR ≥ 0.2 mg/L for tank and point-of-collection samples.

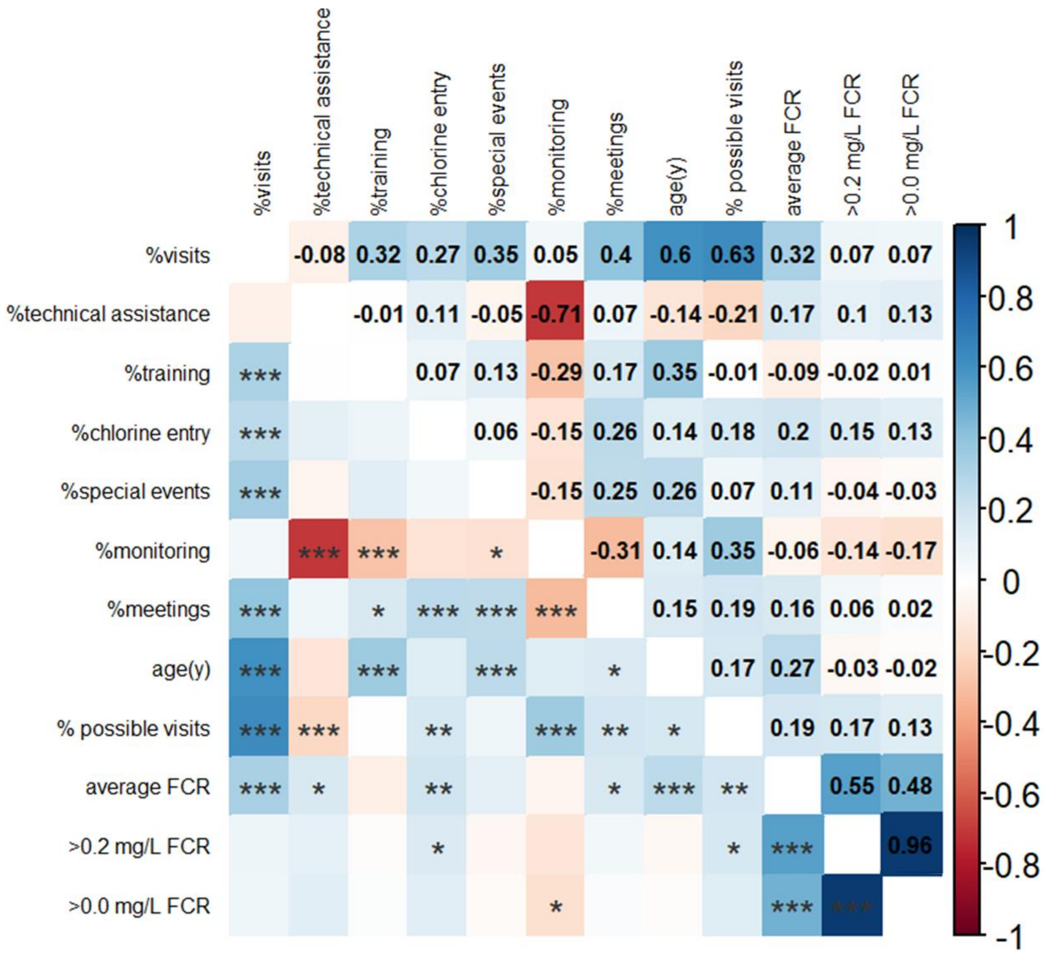

Figure S4. Spearman correlations coefficients (upper triangle) between circuit rider visit periodicity, support types, and average FCR. Adjusted p-values: *****< 0.0045, ****** < 0.0009, ******* < 0.000091 (lower triangle).
